# Structural biology of Parkinson’s disease-associated leucine-rich repeat kinase 2 (LRRK2)

**DOI:** 10.1016/j.jbc.2025.110376

**Published:** 2025-06-14

**Authors:** Andres E. Leschziner

**Affiliations:** 1 Department of Cellular and Molecular Medicine, School of Medicine, University of California San Diego, La Jolla, California, USA; 2 Department of Molecular Biology, School of Biological Sciences, University of California San Diego, La Jolla, California, USA; 3 Aligning Science Across Parkinson’s (ASAP) Collaborative Research Network, Chevy Chase, Maryland, USA

**Keywords:** LRRK2, Parkinson's disease, structural biology, cryo-EM, kinase, GTPase

## Abstract

Leucine-rich repeat kinase 2 (LRRK2) has gone, in a little over 2 decades, from a novel gene linked to cases of Parkinson’s disease (PD) in one family to being the main actionable target for PD therapeutics, with several clinical trials targeting it currently underway. While much remains to be understood about LRRK2—including, chiefly, why its increased activity is linked to PD—much has also been learned. One of the areas where our knowledge has increased exponentially in a very short time is the structural biology of LRRK2. The goal of this review is to provide a survey of the current landscape of LRRK2 structural biology with an emphasis on the functional insights that structures have provided.

## A note on this review

This review is intended for an audience interested in the structural biology of PD-linked LRRK2, and on what we have learned from structures about the function of this protein. Readers interested in a broader survey of the LRRK2 field, including key advances in our understanding of its cell biology, should look at some outstanding recent reviews (1, 2).

I have structured this review in sections addressing different aspects of LRRK2’s structural biology (as opposed to its cell biology). My goal was to make these sections as self-contained as possible so that readers can treat them as entries that provide the desired information without the need to read the entire review.

Supporting Figure 1 is a comprehensive list of published LRRK2 structures, from single domains to the full protein. Structures of LRRK1, LRRK2’s closest homolog in humans, are also listed. I note that because the focus of this review is on the main functional (or therapeutic) insights that have been derived from the structures of LRRK2, not all structures listed in Supporting Figure 1 are discussed in the text.

## A brief history of LRRK2 and Parkinson’s disease therapeutics

LRRK2 was first linked to PD in 2002, when a new locus (*PARK8*) was identified in a Japanese family with a genetic form of PD (3). In 2004, two different groups showed that mutations in LRRK2 were responsible for the genetic or familial form of PD (4, 5). Soon after that, two studies reported that the PD-linked mutations in LRRK2 increased its kinase activity (6, 7). This put LRRK2 on the PD therapeutics map as kinase inhibitors could be developed to treat patients suffering from the LRRK2-linked form of familial PD. This interest in targeting LRRK2 for therapeutics increased dramatically in 2018 when a study showed that LRRK2’s kinase activity was elevated in post-mortem samples from patients who had idiopathic PD, that is with no mutations in LRRK2 (8). This meant that LRRK2-specific inhibitors could potentially be used to treat many, if not all forms of PD. This has made LRRK2 the leading actionable target for PD therapeutics.

The best validated substrates for LRRK2’s kinase are Rab GTPases (9). Rabs are members of the Ras superfamily of small G proteins (the same superfamily to which LRRK2’s ROC domain belongs) and are involved in regulating membrane trafficking (reviewed in (10, 11)). Rabs cycle between inactive (GDP-bound) and active (GTP-bound) states, and their interconversion is catalyzed by Guanine Exchange Factors (GEFs) and GTPase Activating Proteins (GAPs) (11). The nucleotide state of a Rab alters the conformation of two structural motifs known as “Switch I” and “Switch II” (reviewed in (12)). These changes, in turn, affect the interactions between Rabs and other cellular components. LRRK2 phosphorylates its Rab substrates in their Switch II region (9), which would block most interactions with other proteins. In cells, LRRK2 phosphorylates a small subset of the more than 60 Rabs present in the human genome: Rab3A/B/C/D, Rab8A/B, Rab10, Rab12, Rab29, Rab35, and Rab43 (13). Readers are referred to reviews covering the history of Rabs (10, 11), and the specifics of those that are substrates of LRRK2’s kinase (1).

Several LRRK2-specific kinase inhibitors have been developed to date, all of which are of “Type I” (specific features of kinase inhibitor types will be discussed in more detail in the section on LRRK2 therapeutics below). The first one, LRRK2-IN-1, was introduced in 2011 (14), but its use has been limited due to low brain penetrance. The current gold standard in the field, MLi-2, is a compound with high selectivity and affinity for LRRK2 introduced in 2015 (15). Most studies that involve chemical/pharmaceutical/therapeutic inhibition of LRRK2 have used this compound. Two of the most recent Type I inhibitors, from Denali Therapeutics, are the related compounds DNL201 and DNL151 (16). The former was shown to inhibit LRRK2 in PD patients (17) and the latter, renamed as BIIB122, recently entered a Phase 2a clinical trial (clinicaltrials.gov). Although no LRRK2-specific Type II kinase inhibitors have made it to clinical trials yet, the first compounds with good selectivity and potency were recently introduced (18).

At the time of writing this review, there are four clinical trials underway targeting LRRK2. Two of them are for kinase inhibitors—one in Phase 2 for NEU-411 from Neuron23 and the other in Phase 2a, for BIIB122 (mentioned above) (clinicaltrials.gov). A Phase 1 clinical trial is underway in the Netherlands for a PROTAC (ARV-102 from Arvinas), a molecule that targets LRRK2 for degradation by recruiting an E3 ligase. Another Phase 1 trial is evaluating an anti-sense oligonucleotide (ASO) (BIIB094 from Biogen and IONIS Pharmaceuticals) targeting the LRRK2 mRNA (clinicaltrials.gov). Despite the importance of LRRK2 as a target for PD therapeutics, and the number of kinase inhibitors that have been developed, the first structures of LRRK2 bound to inhibitors were only published in 2023 (19) and 2024 (20). These are discussed in more detail below.

## LRRK2: An introduction to the protein

**Figure 1:**
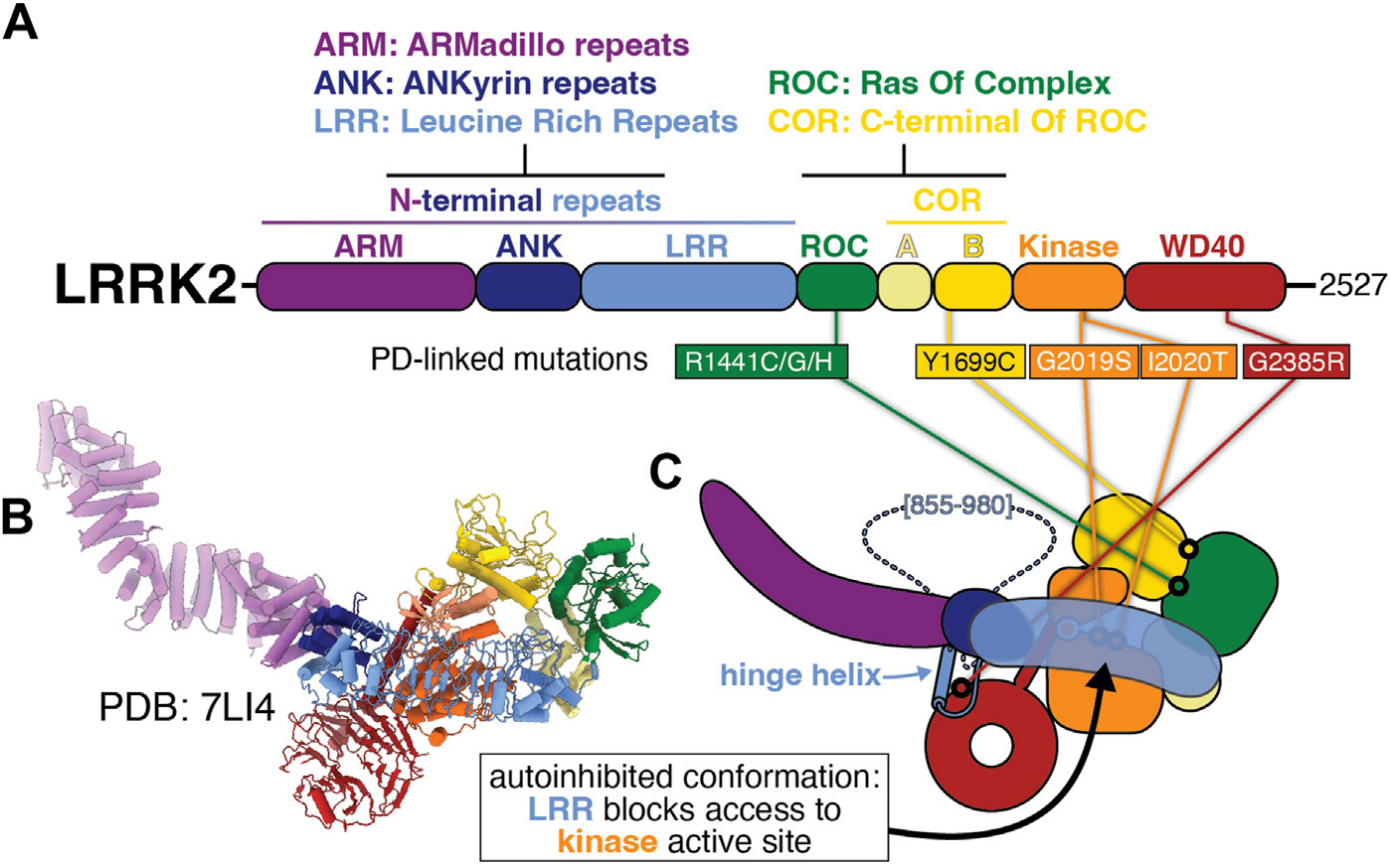
**Domain architecture and overall structure of LRRK2 in its autoinhibited conformation**. *A*, domain architecture of LRRK2, with the most common Parkinson’s Disease (PD)-linked mutations indicated. *B*, *C*, cryo-EM structure (PDB:7LI4) (*B*) and cartoon representation (*C*) of full-length LRRK2 in its autoinhibited conformation. The location of the PD mutations listed in (*A*) are shown in the context of the cartoon. The *dotted line* in (*C*) represents a loop (amino acids 855–980) that connects the hinge helix to the N-terminus of the leucine-rich repeats and has so far been disordered in all published structures. The same cartoon and coloring scheme are used throughout this review.

LRRK2 is a 2527 amino acid multidomain protein containing two enzymatic activities: a Ras-family GTPase and a Ser/Thr kinase (Fig. 1*A*). The N-terminal half of LRRK2 is comprised of repeats: Armadillo (ARM), Ankyrin (ANK), and Leucine-Rich Repeats (LRR), the latter giving rise to the protein’s name (Fig. 1*A*). The C-terminal half contains the GTPase domain, called Ras Of Complex (ROC), the architectural C-terminal Of Roc (COR) domain, with COR-A and COR-B subdomains, the kinase, and a WD40 domain (Fig. 1*A*). The last portion of the protein, following the WD40 domain, is a long alpha helix that lines the back of the kinase, interacting with both its C- and N-lobes (these kinase features are discussed in more detail later). The presence of the ROC-COR motif places LRRK2 in the ROCO family of proteins (21, 22).

Figure 1, *B* and *C* introduces the overall structure of LRRK2 in its autoinhibited conformation, along with a cartoon representation of this structure that is used throughout this review. Two major features of this structure are worth highlighting here (1): the C-terminal half of LRRK2 is J-shaped, thus bringing the GTPase (ROC) in close spatial proximity to the kinase despite their distance along the primary sequence of the protein (2, 23). The LRR domain, which connects to the GTPase, drapes over the kinase, physically blocking access to the active site by its Rab substrates (24). “Undocking” of the N-terminal repeats, which contain the LRR domain, from this position is thought to be necessary for activation of LRRK2. This autoinhibited conformation is stabilized by two sets of interactions; one between the ANK domain and the C-terminal helix on the back of the kinase, and another where the “hinge” helix, which comes out of the LRR, binds to both the junction between the ARM and ANK repeats, and the WD40 (Fig. 1*C*) (24).

Mechanistically, the GTPase domain of LRRK2 remains poorly understood. A number of studies over the years have shown that there is crosstalk between the kinase and the GTPase, and that the GTP-bound state of the latter correlates with increased Rab phosphorylation (25, 26, 27, 28, 29, 30, 31, 32). Despite these insights, and the potential role of the GTPase domain in regulating LRRK2, how its nucleotide state affects the rest of LRRK2 is not understood at a structural or mechanistic level. So far, all published structures of LRRK2 that were solved at resolutions high enough to determine the nucleotide state of the GTPase have been bound to GDP.

Figure 1, *A*–*C* also highlights the most common PD-linked mutations in LRRK2, which are all located in its C-terminal half. As mentioned earlier, all PD-linked LRRK2 mutations are dominant gain-of-function and increase kinase activity. The largest cluster, with four mutations, is at the interface between the GTPase (the ROC domain) and COR-B: three of these mutations affect R1441 (R1441C/G/H), in the ROC domain, while the fourth, Y1699C, is in COR-B, facing the ROC domain. There are two mutations in the active site of the kinase: G2019S and I2020T. G2019 is part of the catalytic DYG triad, and the G2019S mutation is the most common PD-linked mutation in LRRK2. Interestingly, despite their adjacency, LRRK2[G2019S] and LRRK2[I2020T] behave differently in several biochemical and cell biological assays (33) as well as in their clinical features (34).

An important regulatory region in LRRK2, which was disordered in all the structures solved to date, is a loop comprising residues 855 to 980 (Fig. 1*C*). This loop contains several phosphorylation sites that have been shown to regulate its interaction with 14-3-3 proteins (35), an interaction that is believed to keep LRRK2 in its inactive state in the cytosol (35, 36). Loss of phosphorylation in this loop is seen in patients with PD (37).

**Figure 2:**
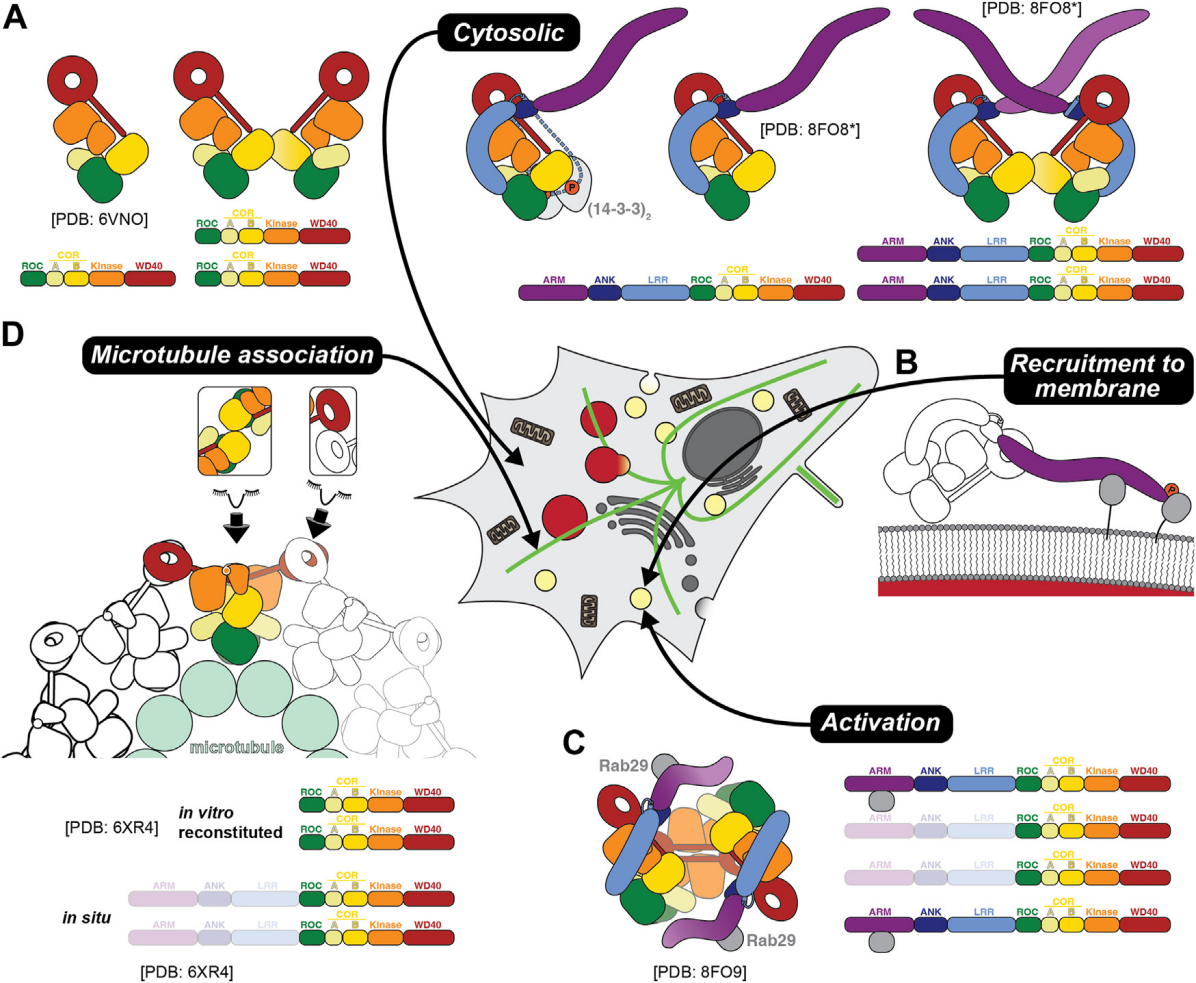
**Summary of the LRRK2 structures, and structural information, discussed in this review**. This figure shows the structures discussed in this review in cartoon representation, following the scheme introduced in Figure 1. In each case, the constructs used in the structural work are shown below the cartoon of the structure. *Dim colors* indicate regions that were present in the construct(s) but were not seen in the structure. The *arrows* indicate the expected cellular localization of the different species. *A*, structures of cytosolic LRRK2. The first structures solved were those of the monomer and dimer of the C-terminal half of LRRK2 (LRRK2^RCKW^, where “RCKW” stands for the four domains present in the construct) (*left side*), followed by those of the full-length protein (*right side*). Most recently (*center*), a structure of full-length LRRK2 bound to a dimer of 14-3-3 was reported. *B*, structural basis for the recruitment of LRRK2 to membranes. Although no structures exist of membrane-associated LRRK2, structural and functional data has shown that LRRK2 is recruited to membranes by interacting with Rab-GTPases *via* recruitment binding sites located in its ARM repeats domain. *C*, structure of a Rab29-bound LRRK2 tetramer proposed to represent an activation species. *D*, LRRK2 can associate with microtubules under certain conditions. This cartoon represents the structure of the microtubule-associated LRRK2 filaments, which have a similar architecture whether reconstituted *in vitro* or formed in cells. The insets above the cartoon eyes highlight the two LRRK2 interfaces involved in forming the filaments: COR-B:COR-B (*left*) and WD40-WD40 (*right*).

In solution, LRRK2 is observed as a mixture of monomers and dimers (reviewed in (38)), the latter being mediated by an interaction between the COR-B domains (Fig. 2*A*). Under certain conditions, LRRK2 can interact with microtubules and form filaments on them; these filaments, which are discussed below, are mediated by both the COR-B interface of the dimer, and an additional interface involving the WD40 domain (Fig. 2*D*). The latter dimer interface had previously been observed in a crystal structure of the isolated WD40 domain (39). However, in the absence of microtubules, the WD40-mediated dimer has only been reported once for the C-terminal half of LRRK2 (referred to as “LRRK2^RCKW^“ for the initials of the four domains that comprise it) under high protein concentrations used for cryo-EM (23), suggesting that it may be weaker than the COR-B:COR-B interface. Although the first structure reported for any portion of LRRK2, a crystal structure of its GTPase (ROC) domain, suggested that it mediated dimerization (40), a ROC:ROC interaction has not been observed in any reported structure of LRRK2^RCKW^ or full-length LRRK2.

## LRRK2 in the cell

LRRK2 is now understood to play a central role in maintaining lysosomal homeostasis and regulating autophagy (see (1, 2) for recent reviews on the cell biology of LRRK2 discussed in this paragraph and the next). LRRK2 becomes activated and recruited to endolysosomal membranes under conditions of stress, such as exposure to lysosomotropic compounds like chloroquine or LLOME. Once at the membrane, LRRK2 phosphorylates Rab GTPases, primarily Rab8A and Rab10. These phosphorylation events trigger a series of downstream responses, including the emergence of tubular structures from lysosomes, secretion of lysosomal content, and membrane remodeling—features thought to support lysosomal repair and recycling. In glial and immune cells such as astrocytes and macrophages, LRRK2 activity is also linked to engagement of the ESCRT-III machinery and calcium-dependent membrane repair, suggesting a broader role for LRRK2 in responding to lysosomal damage.

In addition to supporting repair functions, LRRK2 appears to negatively regulate the degradative capacity of lysosomes and restrict autophagic flux. Mechanistically, this occurs through suppression of lysosomal biogenesis pathways, in part by reducing the nuclear localization of transcription factors like TFE3. LRRK2 also reduces the activity of the lysosomal enzyme glucocerebrosidase (GCase), likely *via* Rab10 phosphorylation. Inhibition of LRRK2 reverses these effects, restoring both GCase function and lysosomal degradation, and reducing α-synuclein accumulation in models of Parkinson’s disease. While Rab29 has been implicated as an upstream activator of LRRK2, its involvement appears to be context-dependent and is not fully understood. Nonetheless, studies in both cell lines and patient-derived neurons show that LRRK2 kinase inhibition can restore lysosomal and autophagic functions, underscoring the therapeutic relevance of these pathways.

The bulk of LRRK2, around 75% as reported in one study (41), appears to exist in the cytosol, likely in its inactive form. This population may be stabilized by interactions with 14-3-3 proteins, in a manner dependent on the phosphorylation of residues S910 and S935 in the disordered 855 to 980 loop. A recently reported structure of LRRK2 bound to a dimer of 14-3-3 proteins (its γ isoform) provided evidence for this (42). In this structure, the 14-3-3 dimer is bound to the COR-A and -B subdomains, with one 14-3-3 monomer engaging each (Fig. 2*A*, middle) (42). While the 855 to 980 loop is still disordered in this structure, densities for phosphoserine are seen bound to 14-3-3, consistent with the role of pS910 and pS935 in mediating 14-3-3 association with LRRK2 (Fig. 2*A*, middle) (42). By associating with COR-A/B while also binding to the 855 to 980 loop, 14-3-3 would constrain the conformation of the N-terminal repeats of LRRK2, whose undocking is thought to be required for LRRK2 activation (42). Intriguingly, binding of 14-3-3 to LRRK2 in this manner would prevent COR-B-mediated dimerization of the latter; what functional role LRRK2 dimerization plays in cells remains an unsolved mystery.

Most of the active pool of LRRK2 is likely to be present on membranes, where its Rab substrates are found (Fig. 2, *B* and *C*) (9). Although the recruitment of LRRK2 to membranes (next section) is one of the better-understood aspects of LRRK2 function, no structures exist of membrane-associated LRRK2.

LRRK2 has been shown to bind to microtubules under overexpression conditions, an interaction that is enhanced by several of the most common PD-linked mutations (33, 43), and by treatment of cells with Type I kinase inhibitors that target LRRK2 (36). It remains to be shown whether this interaction, discussed in more detail later, happens under physiological conditions.

## Recruitment of LRRK2 to membranes

**Figure 3:**
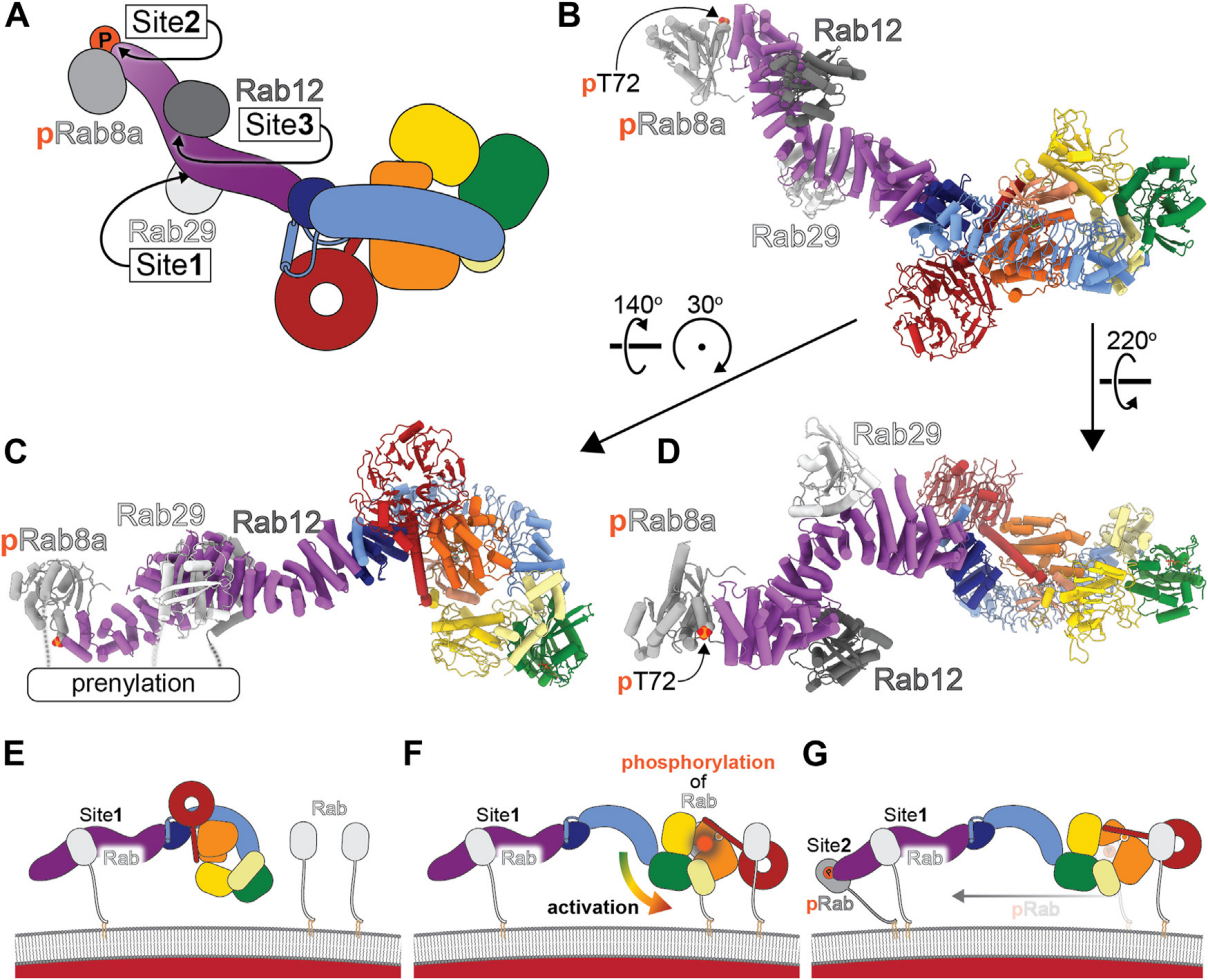
**Recruitment of LRRK2 to membranes**. *A*, *B*, cartoon representation (*A*) and model (*B*) of LRRK2 with recruiting/regulatory Rabs (Rab8a, Rab12, and Rab29) bound to Sites 1, 2, and 3 in its ARM repeats domain. Note that the Rabs shown in *panels* (*A*-*D*) are based on structural data (Rab29) or AlphaFold predictions (Rab8a and Rab12) and do not necessarily represent the specific Rabs responsible for these functions in the cell. *C*, *D*, additional views of the model in (*B*) indicating the locations of the C-terminal ends of the Rabs, which connect them to the membrane-associated prenylation (*C*) and of the phosphorylated Thr72 in pRab8a/10, which increases its binding to Site 2 (*D*). *E*-*G*, model for recruitment, activation, and strengthening of recruitment of LRRK2 to membranes. *E*, LRRK2 is recruited to the membrane through binding of a membrane-associated Rab to Site 1 in the ARM repeats domain. *F*, release of autoinhibition, a process that is not understood, makes LRRK2’s kinase accessible to Rabs as substrates, enabling their phosphorylation. *G*, Phosphorylated Rab binds to Site 2 with higher affinity than the unphosphorylated form. Binding of Rabs to both Site 1 and Site 2 reinforces the recruitment of LRRK2 to the membrane. Although Rab12 has been shown to be a strong regulator of LRRK2, it is not included in the model in *panelsE*-*G* because its role is not yet understood mechanistically.

Rab GTPases are the best-validated LRRK2 substrates (9, 13). To perform its kinase function, LRRK2 must get to the membranes where those Rabs are found. The recruitment of LRRK2 to membranes is one of the better-understood aspects of LRRK2’s activity at the structural level. Work using AlphaFold predictions identified two binding sites for Rab8 and Rab10 in the ARM domain at the N-terminal end of the repeats (44). Interestingly, these are not substrate-binding sites but rather recruitment sites. One site, termed Site #1, located roughly halfway into the ARM domain (residues 360–450 (44)), binds to Rabs unphosphorylated in their Switch II region, the site of LRRK2 phosphorylation (Fig. 3, *A*–*D*). Another site, called Site #2, is located very near the N-terminus of LRRK2 and has higher affinity for Rabs that are phosphorylated in their Switch II region (Fig. 3, *A*–*D*). Residues K17 and K18 are both necessary for this binding (44). The identification of these binding sites led to a model for the recruitment and retention of LRRK2 on membranes that was validated using an *in vitro* reconstituted system and single-molecule total internal reflection fluorescence (TIRF) microscopy (44). In this model, LRRK2 is initially recruited to a membrane by binding to an unphosphorylated Rab through Site #1 (Fig. 3*E*). After this initial recruitment, and once autoinhibition has been released (a process that is not yet understood at a mechanistic or structural level) (Fig. 3*F*), LRRK2 can begin phosphorylating Rab substrates. As phosphorylated Rabs (pRabs) accumulate on the membrane, they bind to Site #2, thus strengthening the tethering of LRRK2 to the membrane (Fig. 3*G*).

More recently, Rab12 was identified as a particularly potent activator of LRRK2’s activity in cells (45). Rab12 binds to yet another site in the ARM domain, Site #3 (Fig. 3, *A*–*D*); as is the case for Sites #1 and #2, Site #3 is also a recruitment site rather than a substrate-binding one. Mutation of either E240 or S244, which are part of the predicted interface between Rab12 and the ARM domain, significantly reduced Rab10 phosphorylation in cells (45). What role Rab12 plays in LRRK2 recruitment and/or activation at a mechanistic level is not yet understood. All three Rab recruitment sites within the ARM domain of LRRK2 engage their Rab partners in the GTP-bound conformation (44, 45). This mode of binding involves interactions with the Switch II region of the Rab, effectively shielding the phosphorylation site—typically a threonine or serine residue—from access by LRRK2. It should be noted that the binding of Rab’s to the recruitment sites #1, #2, and #3 would not interfere, structurally, with the autoinhibited conformation of LRRK2, and that these sites are located far away from both the kinase and GTPase domains. Therefore, if those interactions are also involved in LRRK2’s activation (*i*.*e*.*,* relief of autoinhibition), they would accomplish that through structures that have not yet been captured. Similarly, a structure of LRRK2 bound to a Rab substrate has not yet been published.

Although a model for how LRRK2 is recruited to membranes is emerging, as discussed above, little is known about the subsequent steps. *Is LRRK2’s autoinhibition released before*, *while*, *or after it is recruited to a membrane?* Structurally, all these options are possible; since the recruitment Sites #1, #2, and #3 are in the ARM domain, far away from the C-terminal catalytic half of LRRK2, there is no obvious requirement for autoinhibition to be released before recruitment. Structures of LRRK2 on Rab-containing membranes will be required to answer these questions.

## LRRK2 activation

**Figure 4:**
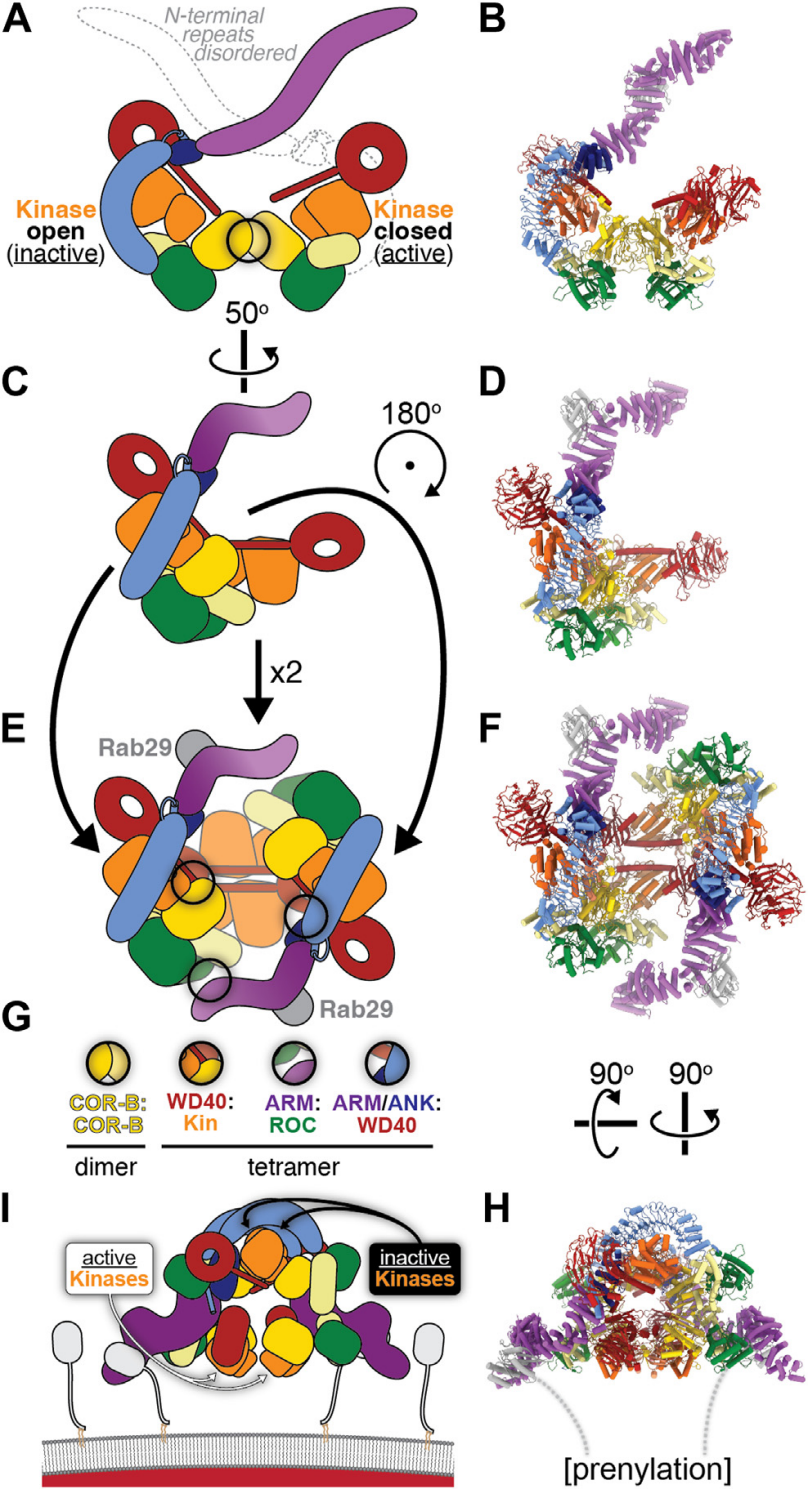
**Structure of a Rab29-bound LRRK2 tetramer containing active LRRK2s at its center**. This figure shows, using cartoons (*left*) and structures (*right*) the architecture of the tetramer. *A*, *B*, The tetramer is a dimer of dimers, where each dimer contains one LRRK2 in its autoinhibited conformation (as shown in Fig. 1), and one LRRK2 in the active conformation. In the latter case, activation is associated with the undocking of the N-terminal repeats, which become flexible and are not seen in the structure, and the closing of the kinase. *C*, *D*, the structures in (*A*, *B*) are rotated to the orientation they will have in the representation of the tetramer. *E*, *F*, the second dimer in the tetramer is related to the one in (*C*, *D*) by a 180^°^ rotation on the plane of the page. *G*, the interfaces involved in the formation of the dimer (*A*, *B*) and tetramer (*E*, *F*). All interfaces are highlighted with *black circles* in the corresponding cartoons: The dimer interface (COR-B:COR-B) in (*A*) and the WD40:Kin, ARM:ROC, and ARM/ANK:WD40 in (*E*). *I*, *H*, these *panels* show how the tetramer might be positioned relative to the plane of the membrane given the orientation of the Rab29 molecules bound to it. The position of the kinase active sites (in both the active and inactive monomers) is indicated.

Although it is clear from our current structural understanding of LRRK2 that the N-terminal repeats must undock for substrates to access the kinase’s active site, how this happens is not known. The first structural evidence for the undocking of the repeats came from the cryo-electron tomography (cryo-ET) reconstruction of microtubule-associated LRRK2 (46). Although full-length LRRK2 had been overexpressed in the cells used to prepare the samples for cryo-ET, the resulting map could only account for the C-terminal half of the protein, suggesting that the N-terminal half—the repeats—were flexible and had been averaged out during sub-tomogram averaging (46). There is only one high resolution structure reported so far that has shown LRRK2 in its active conformation (*i.e*. with the N-terminal repeats undocked) (47) (Fig. 4). This structure, a tetramer of LRRK2 bound to two copies of Rab29, is described next.

Figure 4 outlines the architecture of the Rab29-bound LRRK2 tetramer (47). The structure is a dimer of dimers, where each dimer contains one copy of LRRK2 in its autoinhibited state—that is, with the repeats docked and blocking the active site of the kinase, which is in the open or inactive conformation—and one copy in the activated state (47) (Fig. 4, *A*–*D*). The activated LRRK2 has its kinase in the closed or active conformation, while the N-terminal repeats are not seen in the map, in agreement with what had previously been seen in the cryo-ET of microtubule-associated LRRK2 in cells (47) (Fig. 4, *A*–*D*). Rab29 is bound to Site #1 in the ARM repeats of the autoinhibited LRRK2s (47) (Fig. 4, *E* and *F*). In the tetramer, the activated LRRK2s are found in the center, while the autoinhibited LRRK2s, bound to Rab29, are in the periphery (47) (Fig. 4, *E* and *F*). The tetramer is stabilized by four interfaces, one that is dimer-specific (the COR-B:COR-B dimerization interface discussed above, see Fig. 2*A*), and three that are specific to the tetramer (47) (Fig. 4, *E* and *F*). The authors proposed that this structure represents the activated form of LRRK2 on membranes (Fig. 4, *E* and *I*), with the outer, autoinhibited LRRK2s involved in the retention of LRRK2 on the membrane, and the inner, activated LRRK2s responsible for Rab phosphorylation (47).

The physiological relevance of the Rab29-bound LRRK2 tetramer remains to be established. Some features of the structure are surprising given its proposed role as the active form of LRRK2 on membranes. First, the COR-B: COR-B interface, which is required to form the dimer (Fig. 4*A*), and thus the tetramer (Fig. 4*E*), is not required for activity; instead, mutations that disrupt this interface have been shown to lead to an increase in Rab10 phosphorylation in cells (33, 48). Second, only one of the three interfaces involved in stabilizing the tetramer—an interaction between the ARM domain of the autoinhibited LRRK2 and the ROC domain of the activated LRRK2—is conserved among vertebrate LRRK2s (49).

## LRRK2 inhibition for PD therapeutics

As mentioned in the introduction, LRRK2 was first recognized as a therapeutic target for treating at least some forms of familial PD as early as 2005 (6, 7), and as a potential target to treat many, if not all, forms of PD in 2018 (8). These findings have turned LRRK2 into the leading actionable target for PD therapeutics, and yet the first structures of LRRK2 bound to kinase inhibitors were only published starting in 2023 (20, 50). Prior structures, which are not reviewed here, had been of model kinases, either related to LRRK2 or engineered to make them more similar to LRRK2.

**Figure 5:**
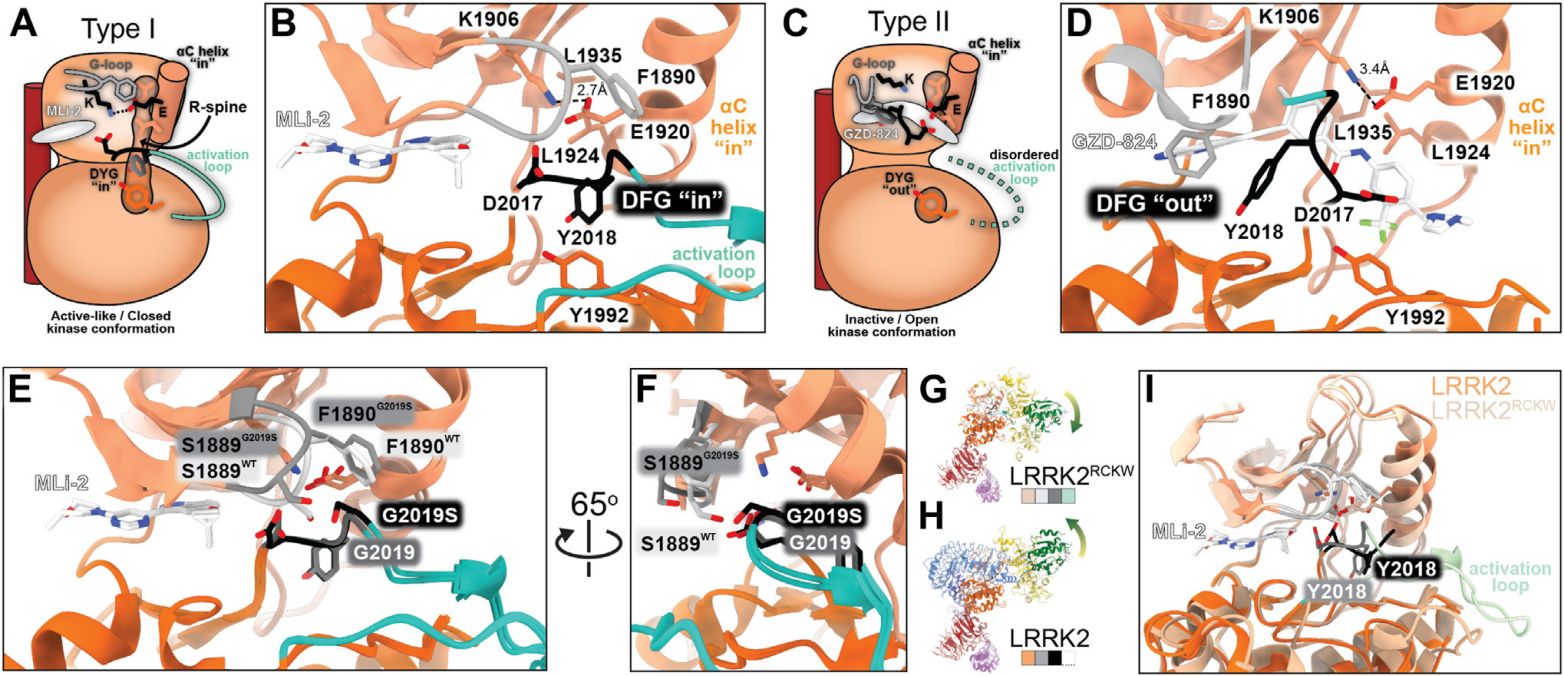
**Structures of LRRK2 bound to kinase inhibitors**. *A*-*D*, structures of LRRK2^RCKW^ bound to Type I (*A*, *B*) and Type II (*C*, *D*) kinase inhibitors. Type I inhibitors trap the kinase in an active-like or closed conformation (*A*) while Type II inhibitors trap it in an inactive or open conformation (*C*). *Panels* (*A*) and (*C*) show the main features of the kinase associated with each type of inhibitor in cartoon form. The *color scheme* is the same used in all *panels* in this figure: the G-loop in *grey*, the catalytic DYG triad in *black*, and the activation loop in *green*. The C-terminal helix of LRRK2, which follows its WD40 domain, is shown in maroon to the *left* of the kinase. *Panels* (*B*) and (*D*) show close ups of the active site of LRRK2^RCKW^’s kinase when bound to either the LRRK2-specific Type I inhibitor MLi-2 (*B*) or the broad-spectrum Type II inhibitor GZD-824 (*D*). All other domains were removed for clarity. *E* and *F*, the G2019S mutation, the most common PD-associated mutation in LRRK2, changes the conformation of the G-loop. The presence of a Ser instead of the usual Gly at position 2019 introduces a clash with S1889 in the G-loop, resulting in a displacement of this residue outwards, away from the DYG motif. All other domains were removed for clarity. *G*-*I*, LRRK2’s autoinhibition prevents the kinase from fully reaching the active-like conformation in the presence of a Type I inhibitor. *G* and *H*, structures of LRRK2^RCKW^ and LRRK2 bound to the LRRK2-specific Type I inhibitor MLi-2. The *yellow/green arrows* highlight how the ROC/COR domains adopt different conformations in the absence (*G*) or presence (*H*) of the docked N-terminal repeats. The colors shown to the *bottom-right* of each structure are those used in *panel* (*I*). *I*, superposition of the kinase domains from the structures in (*G*) and (*H*). All other domains were removed for clarity. Although MLi-2 binds to the kinase in similar ways in both cases, only LRRK2^RCKW^, which lacks the conformational constraint imposed by the docked N-terminal repeats, shows a fully closed kinase and an ordered activation loop. While the DYG motif points inward in both structures, its conformation is different.

The main features of the active/active-like (stabilized by a Type I kinase inhibitor) and inactive (stabilized by a Type II kinase inhibitor) conformations of LRRK2’s kinase are shown in Figure 5, *A* and *C*, respectively, and briefly introduced here (see (51) for a review on kinase structure and (52) for a review of the structural basis of kinase inhibition).

*The active or active-like kinase conformation* (Fig. 5*A*). (i) The two lobes of the kinase come together into a “closed” conformation. (ii) The DYG motif (DFG in most kinases) is positioned in such a way that the catalytic Asp (D2017 in LRRK2) points towards the ATP-binding pocket, where it helps position Mg^2+^ ions, in what is known as the “DYG in” conformation. (iii) This conformation is stabilized by the docking of the Y (or F) residue (Y2018 in LRRK2) into a hydrophobic pocket in the back of the cleft between the two lobes and its stacking, along with residues in both the N- and C-lobes, to form the “R-Spine”, a motif that stabilizes the closed conformation of the kinase. (iv) This also stabilizes the αC helix in its “in” conformation. (v) Sometimes, though not always, this conformation is accompanied by the formation of a salt bridge between a glutamate in the αC helix (D1920 in LRRK2) and a Lys in the N-lobe (K1906 in LRRK2). (vi) Formation of the R-Spine leads to ordering of the activation loop.

*The inactive kinase conformation* (Fig. 5C). (i) The two lobes of the kinase are further apart, in an “open” conformation. (ii) The DYG motif points away from the ATP-binding pocket, in a “DYG out” conformation, because (iii) the Y (or F) residue is prevented from packing into the hydrophobic back pocket by the Type II inhibitor. (iv) The αC helix is still in an “in” conformation; however, (v) the R-Spine is not formed due to the absence of the Y (or F) from the hydrophobic back pocket. (vi) The lack of an R-Spine leads to an unstructured activation loop. (vii) The salt bridge between the Glu in the αC helix and the Lys in the N-lobe is sometimes not formed.

Although several types of kinase inhibitors exist (see (53) for a recent review), only Type I and Type II, the types that have been or are being developed for LRRK2, are discussed here. Type I inhibitors are generally more compact and occupy the ATP-binding pocket. Type II inhibitors are more elongated than Type I, extending beyond the ATP-binding pocket, towards the αC helix. A key feature of Type II compounds, introduced above, is that they block the hydrophobic pocket where the Y (or F) of the DYG motif docks in the active or active-like conformation of the kinase.

Structures have been reported of LRRK2, either full-length or its C-terminal half, bound to several different inhibitors (20, 50). The Type I inhibitors were all LRRK2-specific: DNL201 (20), GNE-7915 (20), LRRK2-IN-1 (20), and MLi-2 (Fig. 5*B*) (50). Since no Type II LRRK2-specific compounds had been reported until very recently, the first structures were obtained bound to broad spectrum Type II inhibitors that show high affinity for LRRK2 and are used in *in vitro* studies: GZD-824 (Fig. 5*D*) (50), ponatinib (20), and rebastinib (20). The structures confirmed the expected Type I or Type II binding modes of the different inhibitors, while revealing their specific interactions. The first LRRK2-specific Type II inhibitor was recently reported in a study that included a structure of LRRK2 bound to it (18). Readers interested in the medicinal chemistry of LRRK2 inhibition are referred to these studies for further details on the structures, though two key findings are discussed here.

An observation with therapeutic potential arose from comparing structures of MLi-2 bound to the C-terminal half of LRRK2 in either its wild-type form or carrying G2019S, the most common PD-linked mutation (Fig. 5, *E* and *F*) (50). The “G-loop” is a Gly-rich loop that connects two β-strands in the N-lobe of the kinase; they are part of a β-sheet that sits on top of the ATP-binding pocket (Fig. 5, *A*–*D*). In the active or active-like states of the kinase this loop curves slightly down, with a Ser at its tip (S1889 in LRRK2) pointing towards the DYG motif (Fig. 5*E*). In the case of the G2019S mutant, the introduction of the Ser creates a clash with the Ser in the G-loop, which results in the latter swinging out, away from the DYG motif (Fig. 5, *E* and *F*). The outward motion of the loop, and the S1889 in particular, creates a small cavity below the G-loop; one might envision an inhibitor that takes advantage of this space and therefore inhibits the PD-linked LRRK2[G2019S] mutant specifically, while leaving the wild-type form unaffected.

Another mechanistically important observation came from a comparison of the structures of MLi-2 bound to either full-length or the C-terminal half of LRRK2 (50). As discussed above, the autoinhibited conformation of LRRK2 stabilizes the kinase in an open/inactive conformation by preventing the movement of the ROC-COR moiety that is required for the kinase to close. By comparing structures of MLi-2 bound to either full-length or the C-terminal half of LRRK2, the authors wanted to determine whether binding of MLi-2 could overcome the inhibitory effect of the N-terminal repeats of LRRK2, either by triggering undocking or by driving conformational changes in the kinase. The structures showed that binding of the inhibitor is not sufficient to overcome the conformational constraints imposed by the repeats (Fig. 5, *G*–*I*). Although the kinase showed some features of the active-like state in the structure of full-length LRRK2 bound to MLi-2 (a “DYG in” conformation and a more closed kinase than is seen in the presence of a Type II inhibitor), key signatures of a canonical active-like state (a fully closed kinase and an ordered activation loop) were still missing and were only observed when using the C-terminal half of LRRK2, which lacks the N-terminal repeats (Fig. 5, *G*–*I*) (50). This suggests that MLi-2, and potentially other Type I inhibitors, could only be engaged in a canonical Type I manner by LRRK2 in its activated state, *i*.*e*.*,* with its N-terminal repeats undocked.

## Microtubule-associated LRRK2

As mentioned above, LRRK2 has been reported to co-localize with microtubules under overexpression conditions (7, 43), an association that is enhanced by most of the common PD-linked mutations (33, 43) and by treatment with Type I kinase inhibitors (36).

**Figure 6:**
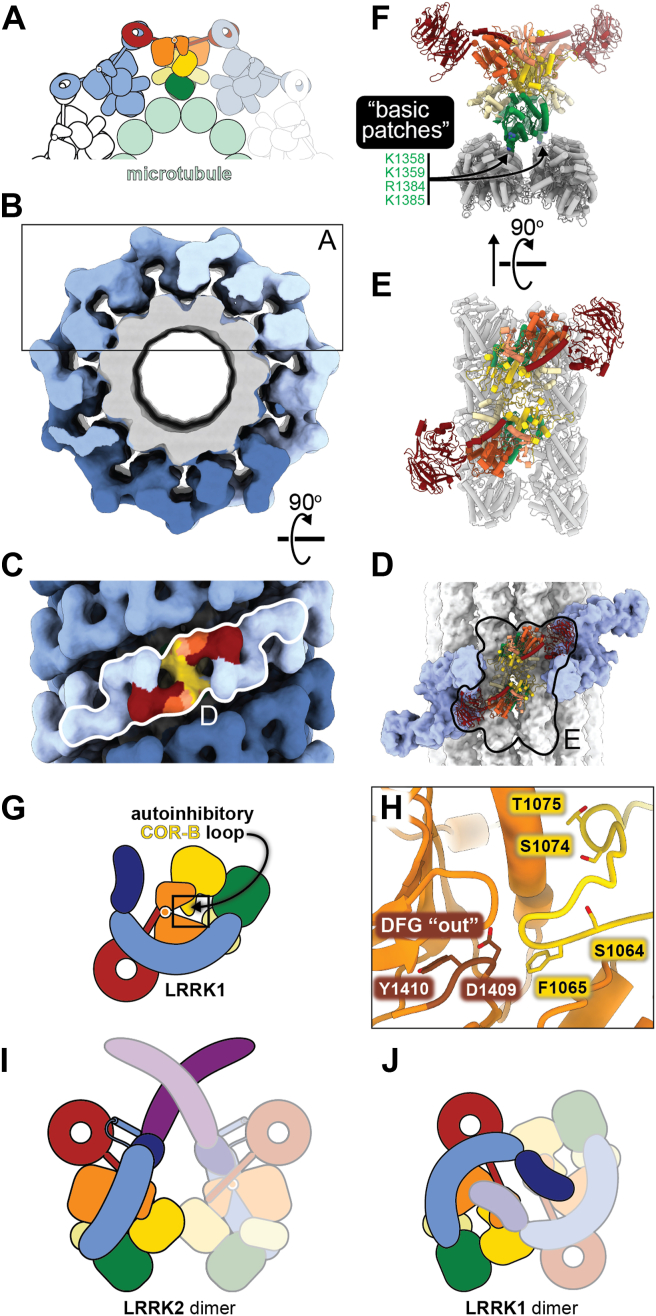
**Microtubule-associated LRRK2 and structure and autoinhibition of LRRK1**. *A*-*C*, cartoon (*A*) and cryo-EM map (*B*, *C*) of in vitro reconstituted microtubule-associated LRRK2^RCKW^ filaments. The box in (*B*) corresponds to the part of the structure represented by the cartoon in (*A*). The three strands of LRRK2^RCKW^ that form the filaments in vitro are shown in different shades of blue. In (*C*), a LRRK2^RCKW^ dimer is shown with the standard domain colors. The *white outline* in (*C*) indicates the region shown in (*D*). *D*, a short segment of a microtubule-associated filament comprised of 3 LRRK2^RCKW^ dimers is shown, with the central LRRK2^RCKW^ dimer in ribbon representation using the standard domain colors, and the neighboring dimers shown in surface representation and colored *blue*. The microtubule, also in surface representation, is *light grey*. The black outline indicates the structure shown in (*E*). *E*, ribbon representation of a LRRK2^RCKW^ dimer on a fragment of a microtubule (two short protofilaments of two tubulin dimers each). *F*, the same structure as in (*E*) but viewed along the microtubule axis. The “basic patches”, short stretches of basic residues that are required for LRRK2’s interaction with the microtubule, are highlighted, and some of the residues are shown in space-filling representation. *G*, cartoon representation of LRRK1, with the same color-coding used for LRRK2. The autoinhibitory loop arising from COR-B is indicated, and the boxed outline highlights the area shown in (*H*). *H*, close-up of the active site of LRRK1’s kinase in its autoinhibited state. The highlighted residues are: the D and Y of the DYG motif; F1065, which prevents Y1410 from docking into the hydrophobic back pocket; and the three residues that are phosphorylated by PKC (S1064, S1074, and T1075). *I*, cartoon representation of the LRRK2 dimer. Note that this representation differs slightly (in terms of the perspective) from the one used in other figures to highlight how LRRK2 and LRRK1 block access to the kinase’s active site in cis or in trans, respectively. *J*, cartoon representation of the LRRK1 dimer.

The first insight into the structure of microtubule-associated LRRK2 came from a reconstruction, using correlative light-electron microscopy (CLEM) and cryo-electron tomography (cryo-ET), of a double-helical filament of LRRK2 wrapped around microtubules from cells overexpressing a GFP-labeled version of the full-length protein containing the PD-linked mutation I2020T (46). Using sub-tomogram averaging, the authors reached a resolution of 14 Å and used integrative approaches to generate a molecular model of the filament (46). Two features of the filaments were particularly striking (1): The LRRK2 density they observed could only accommodate a model of the C-terminal half of the protein, suggesting that the N-terminal repeats were disordered and had been averaged out during processing; and (2) the LRRK2 filaments showed a symmetry mismatch with the microtubules to which they bound. This symmetry mismatch was two-fold. First, the LRRK2 filaments were right-handed while microtubules are left-handed. (Although mathematically a helix can be described with either handedness, the handedness I am using here is the “biological” one that reflects how microtubules assemble.) Second, while microtubules have polarity, the LRRK2 filaments have a pseudo two-fold symmetry axis perpendicular to the microtubule’s axis. This means that the two LRRK2 monomers related by this pseudo two-fold symmetry see different features on the microtubule surface (This symmetry mismatch is illustrated in Fig. 6, which shows a related structure described below.).

This initial reconstruction of the microtubule-bound LRRK2 filaments was soon followed up by the high resolution structure of the C-terminal half of LRRK2 (LRRK2^RCKW^), and the docking of this structure into the 14 Å cryo-ET reconstruction of the filaments (23). In agreement with what the integrative modeling had suggested (46), the structure of LRRK2^RCKW^ could fully account for the microtubule-associated density in the cryo-ET map (23). Docking of the LRRK2^RCKW^ structure into the cryo-ET map also suggested that oligomerization of LRRK2 on the microtubule was dependent on the conformation of its kinase: while the structure of LRRK2^RCKW^, which was solved in the absence of ligand and had its kinase in an open (inactive) conformation, did not dock well into the cryo-ET map, modeling the kinase in a closed (active-like) conformation resulted in a much better fit. This explained why the addition of Type I kinase inhibitors, which stabilize the closed conformation of the kinase, promotes microtubule binding, and led to the prediction that Type II inhibitors, which stabilize the open conformation, would prevent it (23). Using single-molecule biophysics and an *in vitro* system where the molecular motors dynein and kinesin walk along microtubules, the authors showed that the addition of low nanomolar concentrations of LRRK2^RCKW^ blocked the movement of the motors. In agreement with the structural predictions, the addition of Type II kinase inhibitors (the broad-spectrum inhibitors ponatinib and GZD-824) rescued the motors from this inhibitory effect, while Type I inhibitors (the LRRK2-specific MLi-2 and LRRK2-IN-1) exacerbated the effect (23).

The modeling described above was confirmed a couple of years later when a higher resolution cryo-EM structure of *in vitro* reconstituted, microtubule-associated LRRK2^RCKW^[I2020T] filaments was reported (48) (Fig. 6). The structure also revealed the residues in the GTPase domain (ROC) that are involved in the interaction between LRRK2 and microtubules (48). These residues are located in a set of basic “patches” found in the ROC domain (Fig. 6, *D*–*F*) that are conserved among vertebrate LRRK2s but not in other LRRK-family proteins (49). Mutating just two basic residues to Ala significantly reduced the binding of LRRK2^RCKW^ to microtubules *in vitro*, the ability of LRRK2^RCKW^ to block the movement of kinesin along microtubules, and almost entirely abolished the microtubule association of LRRK2 in cells treated with the LRRK2-specific Type I kinase inhibitor MLi-2 (48).

Most recently, a structure of the autoinhibited form of LRRK2[I2020T] bound to a microtubule, where much of the N-terminal repeats can be seen, was determined using *in vitro* cryo-ET (54). Although the interfaces mediating the formation of these filaments were the same as those observed in the structure of activated LRRK2 bound to microtubules (46)—COR-B:COR-B and WD40:WD40—the filaments were left-handed and had a shallower pitch (54).

Despite our growing understanding of the structural basis of the binding of LRRK2 to microtubules, the physiological relevance of this binding remains to be determined. As mentioned above, studies looking at this binding have typically relied on LRRK2 overexpression. An important experiment going forward, due to its therapeutic implications, is to establish whether this microtubule association is observed in cells expressing high endogenous levels of LRRK2 and under conditions that would favor this interaction: LRRK2 carrying PD-linked mutations that enhance microtubule association and treatment with a LRRK2-specific Type I inhibitor.

## LRRK1

Although this review is focused on LRRK2 and its link to PD, I will briefly mention what we have learned about the structure of LRRK1, the closest homolog to LRRK2 in humans, and how the two proteins differ. Unlike LRRK2, LRRK1 is not linked to PD; loss of function of LRRK1 is linked to the rare bone diseases osteopetrosis and osteosclerotic metaphyseal dysplasia (55).

The primary structures of LRRK1 and LRRK2 are very similar, with the former lacking only the ARM domain at its N-terminus. The structures of the LRRK1 and LRRK2 monomers are similar but differ in important ways; while the LRR in LRRK2 lies across and blocks access to the kinase’s active site, it is shifted towards the WD40 domain in LRRK1, leaving the kinase’s active site exposed (49, 56) (Fig. 6*G*). While this might suggest that LRRK1 lacks the autoinhibition present in LRRK2, the opposite is true: LRRK1 is autoinhibited in two mechanistically distinct and coexisting ways. At the level of the monomer, LRRK1 contains a loop in its COR-B domain that extends toward the active site of the kinase (Fig. 6, *G* and *H*). F1065, which sits at the tip of this loop, inserts itself into the hydrophobic back pocket of the kinase, where Y1410 of the DYG motif would normally dock in the “in” or active conformation, thus preventing kinase activation (49) (Fig. 6*H*). This autoinhibition is regulated by phosphorylation of several residues in the same COR-B loop. Three consensus sites for protein kinase C (PKC) are found in this loop: S1064, T1074, and S1075 (Fig. 6*H*). Both phosphorylation of these residues with PKC or phosphomimetic mutations lead to an increase in phosphorylation of Rab7a, a LRRK1 substrate, *in vitro* (56, 57) and in cells (49, 57). In addition to this autoinhibition at the level of the monomer, LRRK1 also sterically blocks access to the active site of the kinase, as LRRK2 does, but it accomplishes this in trans, in the context of its dimer (49, 56) (Fig. 6, *I* and *J*). LRRK1 dimerizes in an antiparallel orientation that shares nothing in common with the LRRK2 dimer; no interface is conserved between the two dimers (Fig. 6, *I* and *J*). In the LRRK1 dimer, the ANK domain of one LRRK1 monomer sits atop the kinase active site of the other monomer, thus preventing access to it by LRRK1’s Rab substrates (Fig. 6*J*). Disruption of dimer-stabilizing interfaces also results in an increase in Rab7a phosphorylation in cells (49).

Why LRRK1 appears to be more tightly regulated, at least at the structural level, than LRRK2, and why LRRK2’s role in PD involves increased kinase activity while the role of LRRK1 in bone diseases involves loss of function is not understood.

## Future prospects

We have learned much about the structural biology of LRRK2 over the last few years. We understand the architecture of the molecule, how it interacts with Rabs that recruit it to membranes, how it binds to kinase inhibitors that could be used to treat PD, and what conformational changes must happen for the protein to be activated (though, importantly, not how those happen mechanistically or in a physiologically relevant setting). I see four areas as the next frontiers in the structural biology of LRRK2. These will give us fundamental insights into how LRRK2 functions in healthy conditions, and how PD-linked mutations alter these functions. At the same time, new structural insights into LRRK2 are likely to reveal new therapeutic targets.

### Visualizing LRRK2 on membranes

*What species represents active LRRK2 on a membrane? What conformational changes take place after LRRK2 is recruited to a membrane?*

### Substrate binding

*How does LRRK2 recognize its Rab substrates?* Despite our growing insight into the recruitment of LRRK2 by Rabs, we do not have a structure of a Rab bound to LRRK2 as a substrate, which would provide important information to understand LRRK2’s substrate specificity.

### Activation/deactivation

Autoinhibition is central to the regulation of LRRK2, with the release of the N-terminal repeats a prerequisite to Rab phosphorylation. *What controls autoinhibition and activation in LRRK2?* The recent structure of LRRK2 bound to 14-3-3 gave us a first look at how autoinhibited LRRK2 can be stabilized in the cytosol. But we do not yet understand what triggers the undocking of the N-terminal repeats of LRRK2, and when that happens in the context of its recruitment to membranes. *What factors*, *including Rabs*, *are involved in LRRK2 activation?* As mentioned earlier, Rab12 is a strong activator of LRRK2’s kinase in cells (45), though the mechanism of this activation remains to be established. Similarly, a mutation (S71R) in Rab32 was recently reported as a risk factor for PD (58, 59). The mutation, which abolishes the phosphorylation site in Switch II targeted by LRRK2, increased LRRK2’s kinase activity in cells (58, 59). It’s becoming increasingly clear that the complexity of LRRK2 as a protein is matched by the complexity of its regulation. Understanding the interplay of all these regulatory factors is a challenge for the coming years.

### Other interactions in the cell

With the exponential growth in interactomics and AlphaFold-driven interaction screens, we will have a growing list of new potential partners for LRRK2. Structural information will be essential in validating these interactions and in potentially providing new therapeutic targets.

## Supporting information

This article contains supporting information.

## Conflict of interest

The authors declare that they have no conflicts of interest with the contents of this article.
