# Supplementary material for: Structural biology of Parkinson’s disease-associated leucine-rich repeat kinase 2 (LRRK2)
Source: J Biol Chem. 2025 Jun 14;301(8):110376. doi: 10.1016/j.jbc.2025.110376 (PMC12337188; doi:10.1016/j.jbc.2025.110376)
Supplement: Supporting Figure [file mmc1.pdf]

PDB	EMDB	Resolution	Description	Cartoon representation of structure	Mutation?	Inhibitor?	Reference
2ZEJ	n/a	2.0Å	ROC domain of LRRK2	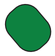			(40)
(no model)	(not deposited)	16.2	LRRK2 dimer	2x 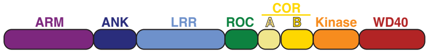			(60)
(no model)	(not deposited)	24.3Å	LRRK1 dimer	2x 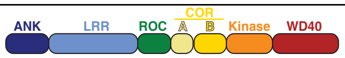			(60)
6DLO, 6DLP	n/a	2.7Å, 4.0Å	WD40 domain of LRRK2	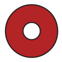			(39)
6VNO	EMD-21250	3.50Å	C-terminal half of LRRK2	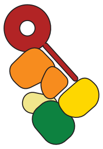			(23)
6XR4	EMD-20825	14.0Å	In situ structure (cryo-ET) of microtubule-bound LRRK2(I2020T) filaments	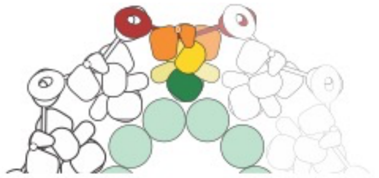	I2020T	Type I (MLi-2)	(46)
7THY,7THZ	EMD-25649, EMD-25658, EMD-25664, EMD-25672, EMD-25674, EMD-25897, EMD-25908	4.5Å to 6.6Å	Microtubule-bound filaments of the C-terminal half of LRRK2(I2020T)	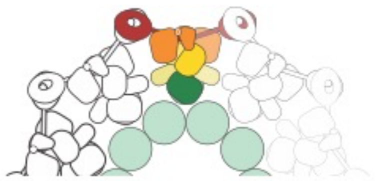	I2020T	Type I (MLi-2)	(48)
7LHT	EMD-23350	3.5Å	LRRK2 dimer	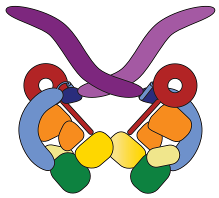			(24)
7LHW	EMD-23352	3.7Å	LRRK2 monomer	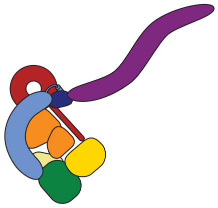			(24)
7LI3	EMD-23359	3.8Å	LRRK2(G2019S) monomer	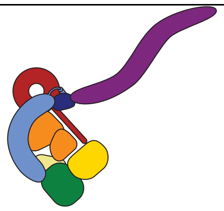	G2019S		(24)
7LI4	EMD-23360	3.1Å	LRRK2 monomer (symmetry-expanded)	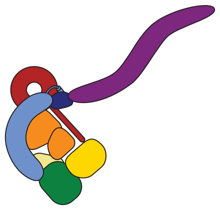			(24)

8E04	EMD-27813	3.8Å	LRRK1 monomer	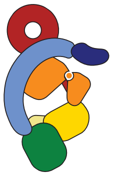			(49)
8E05	EMD-27817	4.6Å	LRRK1 dimer	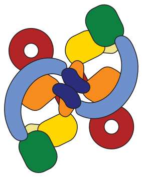			(49)
8E06	EMD-27818	4.3Å	LRRK1 monomer (symmetry-expanded)	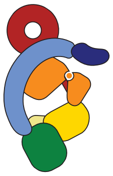			(49)
8FAC	EMD-28950	3.92Å	LRRK1 monomer	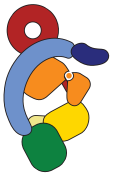			(56)
(no model)	EMD-28952	6.38Å	LRRK1 dimer	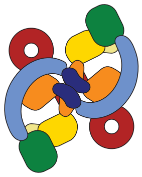			(56)
8FO8	EMD-29341	3.88Å	LRRK2 dimer bound to Rab29	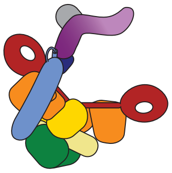			(47)
8FO9	EMD-29342	3.48Å	LRRK2 tetramer bound to Rab29	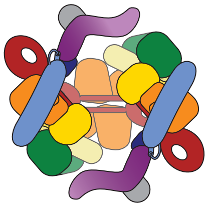			(47)
8SMC	EMD-40588	4.02Å	C-terminal half of LRRK2 bound to type-I inhibitor DNL201	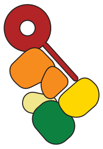		Type I (DNL201)	(20)
8TXZ	EMD-41709	3.05Å	C-terminal half of LRRK2 bound to type-I inhibitor MLi-2	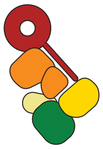		Type I (MLi-2)	(19)
8TYQ	EMD-41728	2.99Å	C-terminal half of LRRK2(G2019S) bound to type-II inhibitor GZD-824	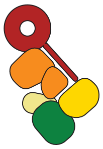	G2019S	Type II (GZD-824)	(19)

8TZB	EMD-41753	3.1Å	C-terminal half of LRRK2(I2020T) bound to type-II inhibitor GZD-824	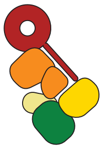	I2020T	Type II (GZD-824)	(19)
8TZC	EMD-41754	2.7Å	C-terminal half of LRRK2(G2019S) bound to type-I inhibitor MLi-2	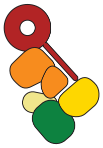	G2019S	Type I (MLi-2)	(19)
8TZE	EMD-41756	2.9Å	C-terminal half of LRRK2 bound to type-II inhibitor GZD-824	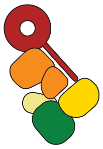		Type II (GZD-824)	(19)
8TZF	EMD-41757	3.4Å	LRRK2(I2020T) bound to type-II inhibitor GZD-824	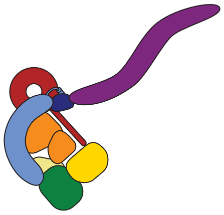	I2020T	Type II (GZD-824)	(19)
8TZG	EMD-41758	2.7Å	C-terminal half of LRRK2(I2020T) bound to type-I inhibitor MLi-2	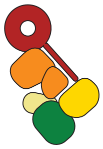	I2020T	Type I (MLi-2)	(19)
8TZH	EMD-41759	3.9Å	LRRK2(I2020T) bound to type-I inhibitor MLi-2	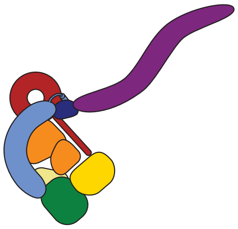	I2020T	Type I (MLi-2)	(19)
8FO7	EMD-29340	3.52Å	C-terminal half of LRRK2 bound to type-I inhibitor LRRK2-IN-1	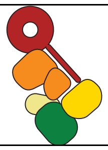		Type I (LRRK2-IN-1)	(20)
8U7H	EMD-41982	3.8Å	C-terminal half of LRRK2 bound to type-I inhibitor GNE-7915	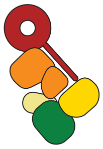		Type I (GNE-7915)	(20)
8U7L	EMD-41985	3.6Å	LRRK2 bound to type-II inhibitor GZD-824	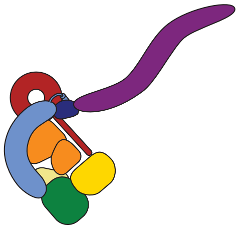		Type II (GZD-824)	(20)
8U8A	EMD-42019	3.4Å	LRRK2 bound to type-II inhibitor ponatinib	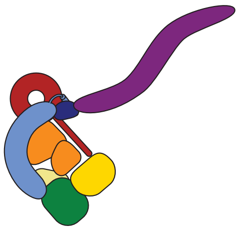		Type II (ponatinib)	(20)

8U8B	EMD-42020	3.7Å	LRRK2 bound to type-II inhibitor rebastinib	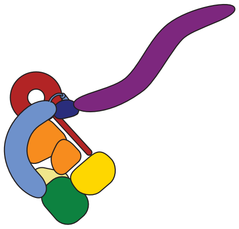		Type II (rebastinib)	(20)
8U1B	EMD-41806	3.66Å	C-terminal half of LRRK2 bound to DARPin E11	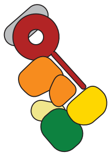			(61)
9CHO	EMD-45591-6	7.8Å – 8.3Å	Structure of autoinhibited LRRK2(I2020T) bound to microtubules	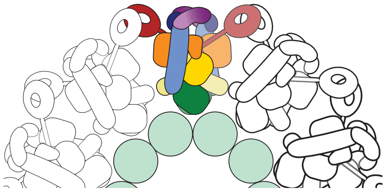	I2020T		(54)
9CI3	EMD-45609	3.96Å	Structure of autoinhibited LRRK2 bound to 14-3-3	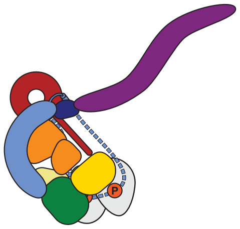			(42)

Supporting Figure 1. List of published LRRK2 (and LRRK1) structures. This table is a comprehensive list of published structures of LRRK2 (from individual domains to the full-length protein) and LRRK1. The columns of the table contain the following information: (1) Protein Data Bank (PDB) accession numbers for the molecular models (when available); (2) Electron Microscopy Data Bank (EMD) accession numbers for cryo-EM maps, when available; (3) reported resolutions; (4) a brief description of the sample; (5) cartoon representation of the structure shown, when possible, in the same orientation used in Figure 2 (or Figure 6 for LRRK1) (for structures where no molecular model is available, the 1D domain architecture is shown); (6) PD mutations, if any, present in the structure; (7) kinase inhibitor, if any, bound to the structure; and (8) the primary citation where the structure was reported.
